# Digit Ratio (2D∶4D) Differences between 20 Strains of Inbred Mice

**DOI:** 10.1371/journal.pone.0005801

**Published:** 2009-06-04

**Authors:** Reginia H. Y. Yan, Mark Bunning, Douglas Wahlsten, Peter L. Hurd

**Affiliations:** 1 Department of Psychology, University of Alberta, Edmonton, Alberta, Canada; 2 Great Lakes Institute, University of Windsor, Windsor, Ontario, Canada; 3 Department of Psychology, University of North Carolina at Greensboro, Greensboro, North Carolina, United States of America; University of St. Andrews, United Kingdom

## Abstract

The second to fourth digit ratio (2D∶4D) is sexually differentiated in a variety of species, including humans, rats, birds, and lizards. In humans, this ratio tends to be lower in males than in females. Lower digit ratios are believed to indicate increased prenatal testosterone exposure, and are associated with more masculinized behavior across a range of traits. The story seems more complicated in laboratory mice. We have previously shown that there is no sex difference in the digit ratios of inbred mice, but found behavioral evidence to suggest that higher 2D∶4D is associated with more masculinized behaviors. Work examining intrauterine position effects show that neighbouring males raise pup digit ratio, suggesting again that higher digit ratios are associated with increased developmental androgens. Other work has suggested that masculinization is associated with lower digit ratios in lab mice. Here, we examine the fore- and hindlimb digit ratios of 20 inbred mouse strains. We find large inter-strain differences, but no sexual dimorphism. Digit ratios also did not correlate with mice behavioral traits. This result calls into question the use of this trait as a broadly applicable indicator for prenatal androgen exposure. We suggest that the inbred mice model presents an opportunity for researchers to investigate the genetic, and gene-environmental influence on the development of digit ratios.

## Introduction

Prenatal androgen exposure is thought to organize many of the male-female differences in the morphology and behavior of both humans and mice alike [reviewed in 1]–[3]. The ratio of the lengths of the second and fourth digits (2D∶4D) is a sexually differentiated trait believed by many to be fixed in utero, and thus, a possible proxy marker for prenatal androgen activity. In humans, males tend to have smaller digit ratios than females [4]–[6], and lower 2D∶4Ds have been associated with more masculine scores on psychological assays such as the Bem Sex Role Inventory [7], [8] and the Buss & Perry Aggression Questionnaire [9], as well as better performance on tests of spatial ability, but poorer performance on tests of verbal fluency [4]. However, meta-analyses have demonstrated inconsistent patterns of results in some of the better known behavioral correlates of digit ratios [10], [11].

Digit ratios are also sexually differentiated in a number of animals, including chimpanzees and gorillas,, wood mice, lizards, and birds [12]–[16]. Male laboratory mice also exhibit lower hind paw digit ratios than females, though this was only found in an outbred strain [17], and in a strain of unspecified genetic composition [18]. No such effect was seen in a larger study of inbred laboratory mice [19], or in the control group of a very large artificial selection study [20]. The direction of the sex effect is also ambiguous. Higher digit ratios are associated with increasing number of male intrauterine neighbours in C57BL/6J mice [21], as well as with more masculine behaviors across strains of lab mice [19]. Since inbred mice are a common model system in fields such as behavioral genetics and endocrinology, it is important to clarify its pattern of variation if digit ratio is to serve as a useful tool to researchers in these areas. Here we examine the sex and strain differences in the digit ratios of 20 inbred mice strains to establish the relationship between digit ratio on the four paws with respect to sex and strain.

## Results

Measurement repeatability (Intra-class correlation [22]) for 2D∶4D was high for all four paws (left front: ICC = 0.87 (95% CI: 0.837–0.898), F_(1,237)_ = 14.5), p<0.0005; right front: ICC = 0.90 (95% CI: 0.879–0.925), F_(1,239)_ = 20.0, p<0.0001; left rear: r = 0.93 (95% CI: 0.906–0.942), F_(1,252)_ = 26, p<0.0001; right rear: ICC = 0.86 (95% CI: 0.827–0.891), F_(1,251)_ = 13.5, p<0.0005). Right front 2D∶4D correlated positively with 2D∶4D on all other limbs; right rear 2D∶4D correlated positively with 2D∶4D on right front and left rear, but not the left front limb (Table 1).

**Table 1:** Correlations of 2D∶4D across limbs.

	Right Rear	Left Rear	Right Front
Left Rear	r_(267)_ = 0.20		
	p = 0.0007		
Right Front	r_(267)_ = 0.17	r_(266)_ = 0.15	
	p = 0.005;	p = 0.013	
Left Front	r_(263)_ = −0.025	r_(262)_ = −0.01	r_(263)_ = 0.17
	p = 0.68	p = 0.82	p = 0.007

All correlations significant at 0.05 level were positive. Right front digit ratio correlated significantly with digit ratio on all other limbs. Right rear digit ratio correlated significantly with digit ratio on right front and left rear, but not left front limbs.

**Figure 1:**
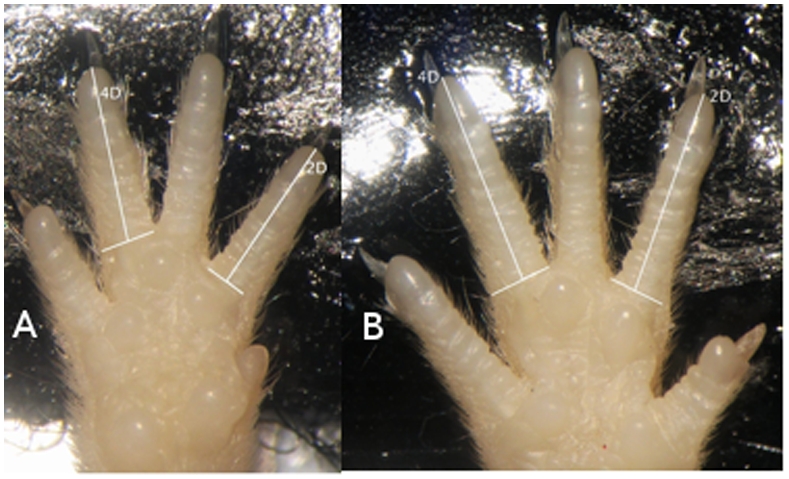
The right front (a) and right rear (b) paw of a C57BL/6J mouse.

**Figure 2:**
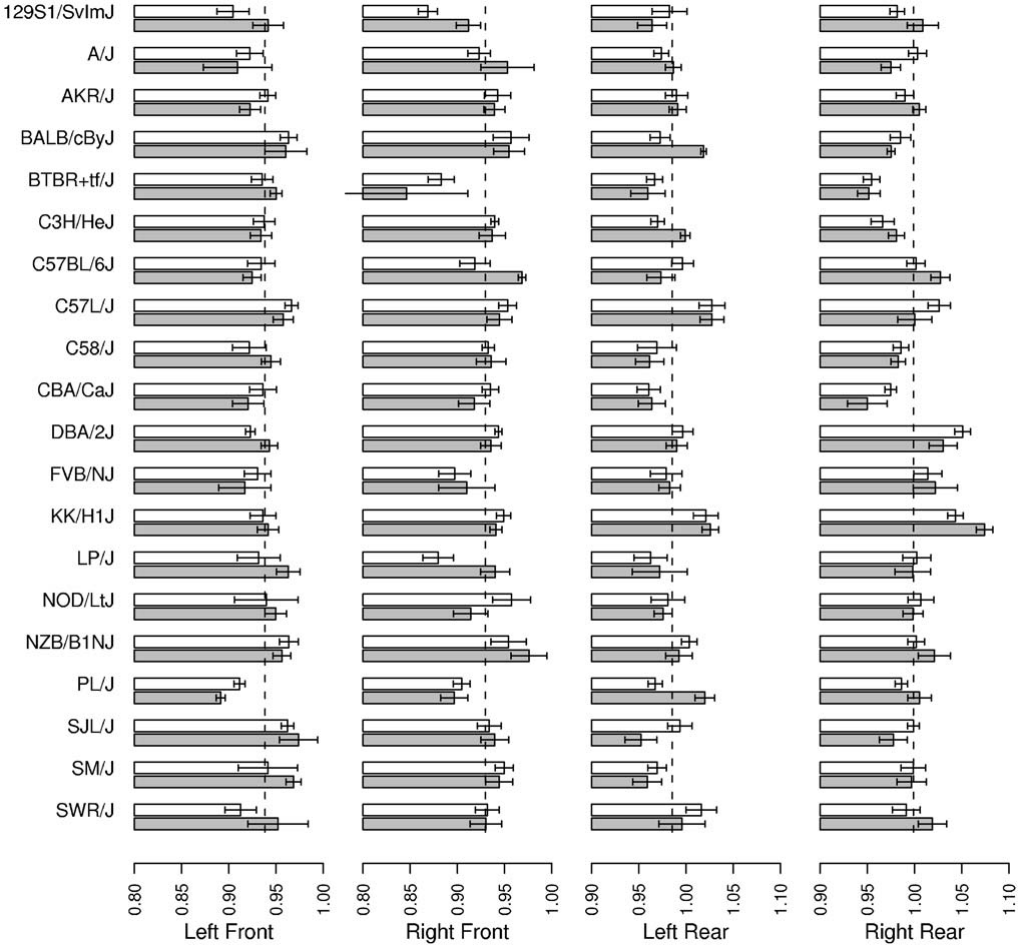
Mean 2D∶4D (±SEM) on each limb by strain and sex. Males are represented by shaded bars, females by open bars. Dotted lines mark the global mean for each paw.

Fore- and hind paws were first analyzed separately, since their morphology is quite different (Fig. 1a and b). Digit ratios on the left side were slightly larger than the right on forelimbs (left: 0.938, right: 0.930, Welch's t_(529.553)_ = 2.39, p = 0.017) while the reverse was true on hindlimbs (left: 0.985, right: 0.999, Welch's t_(536.664)_ = 4.01, p<0.0001). Strain by Sex by Paw ANOVAs revealed significant effects of side (forelimb: F_(1,454)_ = 6.40, p = 0.01; hindlimb: F_(1,459)_ = 22.1, p<0.0001), strain (forelimb: F_(19,454)_ = 5.09, p<0.0001; hindlimb: F_(19,459)_ = 9.73, p<0.0001)), and side-by-strain interactions (forelimb: F_(19,454)_ = 1.71, p = 0.03; hindlimb: F_(19,459)_ = 2.10, p<0.001), but not sex, or any interactions by sex on both fore- and hindlimb digit ratios (p>0.20 for all other effects on both limbs) (Fig 2). When data for each paw were analyzed separately, all four paws showed significant differences between the strains (left front: F_(19,225)_ = 2.27, p = 0.0025; right front: F_(19,229)_ = 4.68, p<0.0001; left rear: F_(19,229)_ = 4.07, p<0.0001; right rear: F_(19,230)_ = 8.19, p<0.0001), but not sex (left front: F_(1,225_ = 1.04, p = 0.31; right front: F_(1,229)_ = 0.46, p = 0.50; left rear: F_(1,229)_ = 0.0031, p = 0.96; right rear: F_(1,230)_ = 0.22, p = 0.64), or sex-by-strain interaction (left front: F_(19,225)_ = 0.74, p = 0.78; right front: F_(19,229)_ = 1.27, p = 0.20; left rear: F_(19,229)_ = 1.16, p = 0.29; right rear: F_(19,230)_ = 1.23, p = 0.23) (Fig 2).

A full repeated measures ANOVA of 2D∶4D on all limbs, with front/rear and left-right as within subjects factors (Table 2) showed strain effects accounted for approximately 36% of between subjects variance, while sex and sex-by-strain effects contributions were not significant. The only consistent within-subjects effects were those related to the difference between front and rear paws, and interactions between this effect and the left/right side effect, and the strain effects. There was no significant interaction effect involving sex.

**Table 2:** Results from a repeated measures analysis of variance on digit ratio.

A. Between-subjects effects					
Effect	Df	MS	F	P	Est. ω^2^
Strain	19	0.0113	8.24	<0.000001	0.26
Sex	1	0.0021	1.51	0.22	nm
Strain × Sex	19	0.0014	1.02	0.43	nm
Within	224	0.0014			
**B. Within-subjects effects**					
**Effect**	**df**	**MS**	**F**	**P**	
Front/Rear (FR)	1	0.802	601.4	<0.00001	
FR × Strain	19	0.006	4.4	<0.00001	
FR × Sex	1	0.001	0.6	0.46	
FR × Strain × Sex	19	0.002	1.3	0.18	
FR within	224	0.0013			
Left/Right (Side)	1	0.0019	1.5	0.22	
Side × Strain	19	0.0025	2.0	0.01	
Side × Sex	1	0.0000	0.01	0.91	
Side × Strain × Sex	19	0.0013	1.1	0.39	
Side within	224	0.0013			
FR × Side	1	0.0314	25.8	<0.00001	
FR × Side × Strain	19	0.0022	1.8	0.02	
FR × Side × Sex	1	0.0001	0.1	0.74	
FR × Side within	224	0.0012			

Within subject factors were front vs. rear and left vs. right. Between strain effects account for approximately 36% of between subjects variance, while sex and sex-by-strain effects contributions are not meaningful (nm). Within-subjects effects significant at the 0.05 level were those difference between front and read paws, and the interactions between this effect and the strain, and between this effect and the left/right side effect; and interactions with strain effects. Sex effects showed no trends towards significance.

The ranking of strain differences across the four limbs was in general agreement for both males (Kendall's W = 0.763, p<0.01) and females (Kendall's W = 0.81, p<0.01). The correlation across strains between male and female 2D∶4D was strong and positive on the right rear limb (r = 0.73, p<0.001) and moderate, but statistically significant on the other three limbs (left rear: r(18) = 0.48, p = 0.03, right front: r(18) = 0.55, p = 0.01, left front: r(18) = 0.52, p = 0.02).

**Figure 3:**
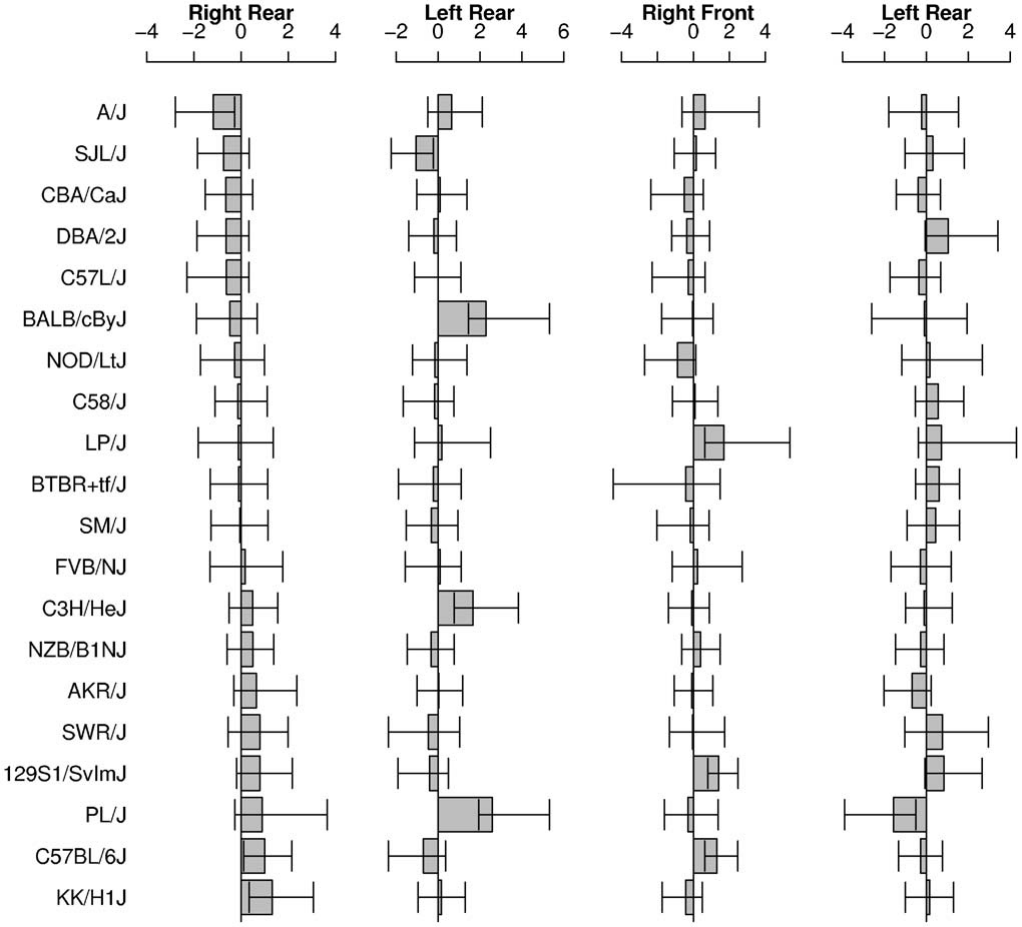
Effect sizes of sex difference in 2D∶4D by strain, a) Right rear paw, b) Left rear, c) Right front, and d) Left front. Shaded bars show the calculated d' sex difference, positive d' values indicate male 2D∶4D greater than female 2D∶4D, negative d' valuess indicate the reverse. Strains are ranked by descending right rear paw effect size. Error bars are 95% confidence intervals calculated from 1000 bootstrap resamplings.

The effect sizes (d' [23]) for sex differences in 2D∶4D on the four limbs are shown in Fig. 3. Positive d' values denote male means larger than female means, negative d' values denote larger female means than male means. We calculated the 95% confidence intervals for each effect size using 1000 bootstrap resamplings [24]. Effect sizes on the right rear limb, where the strain effect was largest, ranged from d' = −1.18 (A/J) to 1.32 (KK/H1J). Of the twenty strains, two (C57BL/6J and KK/H1J) showed 95% confidence intervals on d' entirely above zero (male means were larger than female means) and one (A/J) showed a 95% CI on d' entirely below zero (female means were larger than males). The binomial probability of three 95% confidence intervals excluding zero under the null hypothesis is 0.075. The magnitude and direction of the sex effect sizes on the four limbs showed no general agreement (Kendall's W = 0.005, p>0.05). The number of strains showing sex effects whose confidence intervals did not span zero was 4 on the left rear, 3 on the front right and 2 on the front left. The binomial probability of 12 95% confidence intervals not including zero under the null hypothesis is p<0.001. Of these 12 non-zero including confidence intervals, two were found in the same strain, PL/J, where front left and rear left 2D∶4D appeared to have sex effects in the opposing directions. Of these 12 effects, nine were positive and three negative which is not significantly different from equal, p = 0.15, but shows a trend towards higher 2D∶4D in females than males.

Contrary to the trend noted in [19] —where males tended to have larger 2D∶4D than females in strains where the mean ratio was large and lower digit ratios than females in strains where the mean ratio was small— we found no significant relationship between a strain's mean digit ratio with the direction and magnitude of the difference between the sexes (r_(18)_ = 0.30, p = 0.20). That is, males did not tend to have more extreme digit ratios than females when compared strain-by-strain. However, variance in male hind right digit ratio (var = 0.00187, N = 115) was significantly greater than in females (var = 0.00129, N = 155), F = 1.44, p = 0.03).

Finally, strain mean rear right 2D∶4D did not correlate with inter-strain variation in any of the behavioral traits (total daily activity: males: r_(11)_ = 0.067, p = 0.83; females: r_(10)_ = 0.26, p = 0.42, aggressiveness: males: r_(14)_ = 0.36, p = 0.17; females: r_(14)_<0.001, p = 0.99; anxiety: males: r_(5)_ = 0.16, p = 0.74; females: r_(5)_ = −0.38, p = 0.40), reproductive traits (mice per litter: females: r_(12)_ = −0.13, p = 0.66; % males per litter: females: r_(12)_ = 0.08, p = 0.79), or in body mass(males: r_(18)_ = −0.08, p = 0.74; females: r_(18)_ = −0.05, p = 0.84).

## Discussion

This study demonstrates significant digit ratio variation between mouse strains but not between the sexes. This replicates the major results of [19] in a wider selection of strains. The inter-strain effect was significant on all four limbs, but largest on the right rear paw.

While the lack of sex effect in this study is consistent with the major results of [19], and the lack of sex difference in the control group in [20], it is contrary to two smaller studies of digit ratios in mice [17], [18] — sample sizes: Present study: N = 274; [19] N = 175; [20] Controls N = 428; [17] N = 71; [18] N = 111. Both of these latter two studies found males to have significantly lower digit ratios than females, albeit on different limbs: right rear in [17] and left rear in [18]. Possible explanations for the discrepant sex results between our studies and the two previous ones include: differences between limbs, measurement methods, and differences in the mice due to inbreeding.

That our previous study found no sex effect has been attributed to our use of hind limb digit ratios [25]. The present results show this to be extremely unlikely. None of the four paws exhibited a sex difference in 2D∶4D, nor was the sex effect significant when pooled across all limbs in the omnibus anova (Table 2). All significant correlations (which was 4 of the 6 possible) between limbs were positive, and the ranking of strain differences across all four limbs showed significant concordance. This suggests that the effects seen on right rear 2D∶4D are not qualitatively different from that of the ratios on other limbs. McFadden & Shubel [26] suggested that human 2D;4D ratios were inversely related on the hands and feet. We found no evidence of such an effect in mice.

Our method of 2D∶4D measurement also differs from that of the other studies. Our technique is a direct application of the standard method used on human subjects [5], [6]. On the other hand, Brown et al. [17] measures digit length by using a “pin method”, which is sensitive to the depth of webbing between the digits (see [19] for discussion), while Manning et al., [18] do not specify their methods. We think it unlikely that our technique, which is demonstrably capable of detecting strain effects and correlations across paws, lacks the power to detect a moderately sized sex effect.

We have suggested that inbreeding may somehow influence sex differences in digit ratio [19]. In a study of over a thousand mice, half of which were artificially selected on a behavioral trait, we found no sex difference in the unselected group, while females in the selected group had higher right rear 2D∶4D than did selected males [20]. Brown et al. [17] used an outbred mouse line, while Manning et al. [18] did not describe the source of their mice. Discrepant results, apparently due to large inter-sample variation, are not uncommon when investigating digit ratios in animals. Forstmeier [27] found that behavioral correlates to digit ratio in zebra finches differed significantly between generations in the same captive population. He also found no sex effect in his birds, unlike Burley & Foster [15] who found a significant difference in their captive population of zebra finch. Similarly, Romano et al. [28] found no 2D∶4D sex difference in one strain of ring-necked pheasants, but a significant difference in another strain [16].

A likely explanation for the lack of an overall sex effect in mice 2D∶4D is that males have larger digit ratios in some strains, and smaller ratios in other strains, with many strains showing no reliable difference at all, as suggested by the results in Fig. 3. Two strains (A/J and C57BL/6J) of the three which seemed to show sex differences in 2D∶4D on the right rear limb (where strain effects were largest) were also included in a previous study [19], where they showed non-significant differences in the same direction as in the present study, but in opposite directions to each other (A/J: Welch's t_(20.68)_ = −0.78, p = 0.44, d' 95% CI from −1.17 to 0.47; C57BL/6J: Welch's t_(19.0)_ = 0.80, p = 0.43, d' 95% CI from −0.51 to 1.19). Further investigations into a subset of strains studied here using substantially larger samples will tell us whether this pattern of digit ratios is in fact the case.

We failed to replicate any of the relationships between 2D∶4D and behavioral mice traits seen across strains in [19]. All the mice in our study were raised, housed and tested under identical conditions for their entire lives. If environmentally induced variation in maternal state produces the correlations normally seen between digit ratio and behavioral traits, then the uniform lab environment in which our mice were raised may account for the lack of correlations seen in this study. The inter-strain variation in the digit ratios of our inbred mice suggests that genetic differences also contribute to digit ratio differences. Forstmeier [27] found no sex difference in his zebra finches, but did find additive genetic variation to account for 71–84% of the digit ratio variation in his birds. The very strong ethnic group effect on variation in human digit ratio remains largely unexplained. Manning [4] have suggested that human 2D∶4D is a function of latitude, such that those residing in intermediate latitudes have higher digit ratios than those residing in lower or higher latitudes. Loehlin et al. [29] suggest that this trend does not exist in a larger sample. When the human populations' mean digit ratio is analyzed as a function of another sexually differentiated trait, mean stature, separate regression intercepts emerge for each sex, suggesting that androgen variation cannot be the single mechanism responsible for both the sex and inter-ethnic differences seen in height and 2D∶4D [30]. Given that all our mice were subjected to the same lab environmental conditions and behavioral tests prior to euthanasia, and that we found significant digit ratio variation between strains, but not within strains, it is clear that genetic variations must be a second mechanism that influences the development of digit ratios. The varying direction of sex effect on digit ratio calls into question the use of this trait as a broadly applicable indicator for prenatal androgen exposure.

## Materials and Methods

All animal protocols and procedures were reviewed and approved by the University of Alberta's Biological Sciences Animal Services' ethics review committee (protocol #538705) and the University of Windsor Animal Care Committee (protocol #05-17).

Inbred mice of 20 strains (116 males and 158 females, mean (±sd) 6.9±2.1 mice per sex-by-strain combination, range 3–12) were obtained from Jackson Laboratories (Bar Harbor, ME). This sample size was more than adequate to detect any consistent sex effect, or between strain variation [31]. All were subjected to the same housing conditions and tests of an unrelated study. The mice were euthanized at the conclusion of the study in accordance with all the applicable laws and guidelines as approved by the ethics review committees. Paws were removed after euthanasia and preserved in 10% formalin solution.

Paw photography and digit length measurement were done according to the method of [19]. Paws were placed palm side up onto a piece of adhesive backing to ensure straight digits, and photographed under a microscope. Two photographs of each paw were taken for reliability measurements. Digit length (from the mid-point of the basal crease to the tip of the digit) was measured in pixels using the GNU Image Manipulation Program. Digit ratios were calculated by dividing the length of the second digit by the length of the fourth. Paws with missing or damaged digits were dropped from the dataset, but other paws on the same animal were used. This results in slightly different sample sizes for similar analyses on different paws.

We also assessed the relationships between mean strain 2D∶4D and a number of behavioral traits, such as aggressiveness (the number of bites delivered during testing), anxiety (percent of time spent in an open field test), and total daily activity, as well as with reproductive traits and body weights. Data for these traits (except for body weights) were taken from the Mouse Phenome Database (http://www.jax.org/phenome). The data sets (with trait abbreviations) obtained from the Mouse Phenome Database were: MPD:92 (tot_daily), MPD:149 (mice_per_litter), MPD:149 (per_males_wean), MPD:160 (n_bites), MPD:118 (pct_open).
